# The Colorado Dental Law

**Published:** 1889-08

**Authors:** O. C. French

**Affiliations:** Chief Enrolling Clerk of Senate.


### Editorial, Etc.

---

THE COLORADO DENTAL LAW.—A Bill for an Act to Insure the Efficiency of Practitioners of Dental Surgery and to Regulate the Practice of Dentistry in the State of Colorado. *Be it enacted by the General Assembly of the State of Colorado:*

SECTION I. No person shall practice dentistry in this State until he or she shall have obtained a license for such purpose as hereinafter provided, but nothing in this act shall be construed to prohibit any physician or surgeon from extracting teeth.

SEC. 2. A State Board of Dental Examiners shall be and is hereby created whose duty it shall be to enforce and execute the provisions of this act. The said board shall consist of five members, who shall be appointed by the governor, by and with the advice of the sena'e. Each member of said board shall be appointed for the term of two years, and hold his office until his successors be duly appointed. Vacancies occurring in said board shall be duly filled by the governor.

SEC. 3. Said board shall choose from its members a president, secretary and treasurer thereof, and shall meet at least once in each year, and as much oftener and at such times and places as it may deem necessary. The first meeting of said board shall be held within sixty days after the time this act will go into force and effect, at the capital of the State. A majority of said board shall at all times constitute a quorum, and the proceedings thereof shall at all reasonable times be open to public inspection.

SEC. 4. Any person desirous of continuing the practice of dentistry within this State shall appear in person before said board, at the first or any subsequent meeting of said board, at such time and place as the said board may designate, and submit to an examination by said board, to determine his or her ability to continue the practice of dentistry in this State; provided, however, that the secretary of said board may, upon application of any such person, issue a permit to temporarily practice dentistry until the next meeting of the board, but no longer, unless the board at such meetings shall extend such temporary permit; and such person making application for examination by the board as aforesaid shall deposit with the secretary of said board a fee of ten ($10.00) dollars as compensation for making said examination. All persons who may be found qualified by said board upon any such examination, to continue the practice of dentistry in this State, shall receive from said board a certificate signed by the president, and attested by the secretary, under official seal of said board, authorizing the holder thereof to thereafter continue the practice of dentistry in this State.

SEC. 5. Any and all persons who shall so desire may appear before said board at any one of its meetings, and be examined with reference to their knowledge and skill in dental surgery, and if the examination of any such person or persons shall prove satisfactory to said board, the Board of Examiners shall issue to such persons as they shall find from such examinations to possess the requisite qualifications, a license to practice dentistry in accordance with the provisions of this act.

SEC. 6. Any person who shall violate any of the provisions of this act shall be deemed guilty of a misdemeanor, and shall be liable to prosecution before any court of competent jurisdiction, upon information or by indictment, and upon conviction may be punished by a fine in a sum not less than one hundred ($100.00) nor more than five hundred ($500.00) dollars, or by imprisonment from one to ninety days, or both in the discretion of the court. Each day that this act is violalted shall be considered a separate offence.

SEC. 7. Said board shall be authorized, out of the funds coming into its possession from the fees authorized by this act, to pay to each member thereof such compensation as the board may determine, and all legitimate and necessary expenses incurred in attending the meetings of said board. Said expenses shall be paid only from the fees received by the board under the provisions of this act, and no part of the compensation or other expenses of the board shall ever be charged against or paid out of the State treasury. All moneys received in excess of said expenses shall be held by the treasurer of said board as a special fund for the meeting of the expenses of said board, by giving such bond as the board shall from time to time direct. Said board shall make a biannual report of its proceedings to the governor, by the fifteenth day of December of the year preceding the next ensuing session of the legislature, together with an account of all moneys received and disbursed by them pursuant to this act.

I have compared this copy with the enrolled copy and find it correct.

O. C. FRENCH,
Chief Enrolling Clerk of Senate.

